# Glufosinate-Ammonium Induced Aberrant Histone Modifications in Mouse Sperm Are Concordant With Transcriptome in Preimplantation Embryos

**DOI:** 10.3389/fphys.2021.819856

**Published:** 2022-01-25

**Authors:** Xuan Ma, Yun Fan, Wenwen Xiao, Xingwang Ding, Weiyue Hu, Yankai Xia

**Affiliations:** ^1^State Key Laboratory of Reproductive Medicine, Center for Global Health, School of Public Health, Nanjing Medical University, Nanjing, China; ^2^Key Laboratory of Modern Toxicology of Ministry of Education, School of Public Health, Nanjing Medical University, Nanjing, China; ^3^Department of Microbes and Infection, School of Public Health, Nanjing Medical University, Nanjing, China; ^4^Department of Nutrition and Food Safety, School of Public Health, Nanjing Medical University, Nanjing, China

**Keywords:** glufosinate-ammonium, H3K4me3, H3K27ac, sperm, embryo development

## Abstract

Glufosinate-ammonium (GLA) is a widely used herbicide with emerging concern over its male reproductive toxicity. Abnormalities in sperm histone modification induced by GLA exposure observed in our previous study aroused our interest in whether such alterations could further affect embryonic gene expression. Here we administered adult male mice with 0.2 mg/kg⋅day of GLA for 5 weeks to collect their sperm or 4-cell embryos after copulation. Cleavage Under Targets and Tagmentation (CUT&Tag) sequencing showed alterations of sperm H3 lysine 4 trimethylation (H3K4me3) and histone H3 lysine 27 acetylation (H3K27ac), which are active histone modification marks involved in embryo development, while RNA sequencing identified differentially expressed genes in 4-cell embryos. Differentially H3K4me3 and H3K27ac occupied regions were mainly distributed at the gene promoters and putative enhancers, and were enriched in pathways related to the immune system and nervous system. Integrative analysis of these sequencing data showed that genes such as *Mgl2* with increased H3K4me3 and H3K27ac in sperm were up-regulated in embryos, and *vice versa* for genes such as *Dcn*. Additionally, differentially occupied H3K4me3 and H3K27ac in sperm were linked to gene expression changes in both paternal and maternal alleles of 4-cell embryos. In conclusion, GLA-induced changes in sperm H3K4me3 and H3K27ac are concordant with gene expression in preimplantation embryos, which might further affect embryo development and offspring health.

## Introduction

Histones consisting of H2A, H2B, H3, and H4 are proteins that are critical in the packing of DNA into the cell. Lysine residues in N-terminal tails of histone H3 are the major targets for post-translational modifications (PTMs) such as methylation and acetylation, which are closely associated with gene transcription activities (Yan and Boyd, 2006). Unlike somatic cells, most histones are replaced by protamines for condensed packaging of DNA during spermatogenesis, and only 1% histones are retained in mature mouse sperm (Siklenka et al., 2015). Although limited in quantity, the retained histones in sperm bearing abundant modifications are still essential in male reproduction (Liu et al., 2019), embryo development (Hammoud et al., 2009; Yoshida et al., 2018) and offspring health (Siklenka et al., 2015). Notably, histone H3 lysine 4 trimethylation (H3K4me3) and histone H3 lysine 27 acetylation (H3K27ac) have been well established as active promoter and enhancer marks respectively (Calo and Wysocka, 2013). It is reported that regions marked with H3K4me3 in sperm are mainly enriched at fertility and development related genes, and parts of them can be transmitted into embryos (Zhang et al., 2016; Lismer et al., 2020; Lambrot et al., 2021). Similarly, sperm H3K27ac is highly associated with *in vivo* fertility (Kutchy et al., 2018) and 3D chromatin architecture in embryos (Wike et al., 2021). Therefore, both sperm H3K4me3 and H3K27ac are essential for fertility and embryo development.

Sperm histone modifications are susceptible to environmental factors, such as diet (Carone et al., 2010; Lismer et al., 2021) and chemical exposures (Lombó and Herráez, 2021), leading to potential adverse outcome of offspring health from embryonic to adult stage. For example, sperm H3K4me3 at putative enhancers of developmental genes was altered by a folate-deficient diet, and then transmitted to preimplantation embryos resulting in deregulated embryonic gene expression (Lismer et al., 2021). For chemical exposures, paternal bisphenol A (BPA) exposure increased histone H3 lysine 9 acetylation (H3K9ac) and H3K27ac in zebrafish sperm, and further impaired heart development in progeny (Lombó et al., 2019; Lombó and Herráez, 2021). More findings are required to support the link between environmental factors-induced sperm H3K4me3/H3K27ac alterations and preimplantation embryos.

Glufosinate-ammonium (GLA), one of the most widely applied broad-spectrum herbicides, is highly hydrophilic and considered safe when properly used (Takano and Dayan, 2020). However, emerging evidence suggests its potential toxicity. An *in vivo* study on lizards observed GLA-induced severe testis lesions by oxidative damage (Zhang et al., 2019), while an *in vitro* study on human sperm recently revealed its impairment on sperm mitochondria respiration efficiency (Ferramosca et al., 2021), which indicates GLA toxicity on male reproduction. Our previous study (Ma et al., 2021) also showed its effects on male reproductive health with significantly altered DNA methylation, histone H3 lysine 27 trimethylation (H3K27me3) and histone H3 lysine 9 trimethylation (H3K9me3), as well as transcriptome in mouse sperm. The impact of GLA exposure on sperm histone modifications and transcriptome raised our awareness on its potential risks for embryo development, whereas no other data has shown such the risk to date. Given the role of sperm H3K4me3 and H3K27ac in gene transcription and embryo development, we hypothesized that GLA exposure would affect these two epigenetic marks in sperm, and thus disrupt embryonic gene expression.

In this study, after administration of GLA at a dose of 0.2 mg/kg⋅day to adult male mice for 5 weeks, we performed Cleavage Under Targets & Tagmentation (CUT&Tag) to demonstrate genome-wide H3K4me3 and H3K27ac profiles in sperm, and RNA sequencing (RNA-seq) to display transcriptome in 4-cell embryos derived from GLA exposed male mice. By integrating these sequencing data, we tried to answer whether GLA exposure affects H3K4me3 and H3K27ac profiles of sperm and transcriptome of preimplantation embryos.

## Materials and Methods

### Animals and Glufosinate-Ammonium Administration

C57BL/6J male mice aged 6–8 weeks (purchased from Animal Core Facility of Nanjing Medical University) were housed in a constant environment, and they were randomly divided into two groups either for the control (CON) or GLA treatment after 1-week acclimation. The CON group had free access to ultrapure water, while the GLA group was treated with GLA (purity ≥ 98.0%; Sigma-Aldrich, 45520) through drinking water at a dose of 0.2 mg/kg⋅day, which was equivalent to the acceptable daily intake (ADI) of 0.01 mg/kg (JMPR, 2012) after conversion from human to mice (Nair and Jacob, 2016) and consistent with our previous study (Ma et al., 2021). Glass bottles of drinking water were renewed twice a week to keep the dose constant. After a 5-week administration, these male mice were mated with super-ovulated DBA/2 female mice (purchased from Beijing Vital River Laboratory Animal Technology Co., Ltd.) to obtain 4-cell embryos. GLA administration was continuous except during mating. Male mice were sacrificed for sperm collection once enough embryos were collected for further experiments. All animal procedures here were approved by the Institutional Animal Care and Use Committee (IACUC) of Nanjing Medical University (1811056–2).

### Sperm Collection

After a successful mating within 1 week, male mice (*n* = 10 for each group) were sacrificed to collect mature sperm from bilateral cauda epididymidis as previously described (Sharma et al., 2016; Ma et al., 2021). Then, we counted 1,000 sperm under a microscope, and verified that the sperm purity was over 99.9%. Sperm from five mice were pooled as a biological replicate, and two biological replicates in each group were immediately subjected to the subsequent experiment.

### CUT&Tag on Sperm and Library Preparation

To examine genome-wide H3K4me3 and H3K27ac enrichment on sperm DNA in the nucleus *in situ*, we performed chromatin profiling with CUT&Tag according to its latest protocol (Kaya-Okur et al., 2020). In brief, we permeabilized fresh sperm in ice-cold NE1 on ice for 10 min, followed by light crosslinking with formaldehyde to fix nuclei. Then, sperm nuclei bound to Concanavalin A-coated Magnetic Beads (Novoprotein Scientific Inc., N251). Next, H3K4me3 rabbit pAb (1:100; PTM BIO, PTM-613) and H3K27ac rabbit pAb (1:100; PTM BIO, PTM-116) were employed as primary antibodies for nuclei binding, and Goat Anti-Rabbit IgG H&L (1:100; Abcam, ab6702) was used to bind primary antibodies. After binding pG-Tn5 adapter complex (1:100, Vazyme, S602), tagmentation was conducted, followed by DNA extraction with Phenol:Chloroform. After library preparation by PCR amplification (Vazyme, TD202 and TD601), DNA products were purified with Ampure XP beads (Beckman Coulter, A63881). After DNA quantification and qualification, all libraries were sequenced using NovaSeq, 6000 (Illumina, United States) by Beijing Novogene Bioinformatics Technology Co., Ltd., China.

### CUT&Tag Data Processing

We conducted CUT&Tag data processing according to a step-by-step protocol described previously (Henikoff et al., 2020). In brief, all paired-end CUT&Tag reads after trimming were aligned to the mm10 genome using Bowtie2 v2.4.4 (Langmead and Salzberg, 2012). All unmapped reads, non-uniquely mapped reads and PCR duplicates were removed. Then, peaks and enriched regions typically for CUT&Tag profiling were called with SEACR v1.3 (Meers et al., 2019). Noisy peaks with very weak signals were removed in further analysis.

### 4-Cell Embryo Collection

To induce superovulation, 3–4-week-old DBA/2 female mice were intraperitoneally administered 5 IU of PMSG, followed by injection of 5 IU of hCG 48 h later. After injection of hCG, each female mouse was mated with one CON or GLA mice, and sacrificed 56 h after hCG administration to dissect its oviduct. Then, we flushed 4-cell embryos from oviducts with M2 medium. Embryos from three female mice were pooled as a biological replicate, and three biological replicates were contained in each group (*n* = 9). Embryos in M2 medium (at least 30 embryos in each replicate) were immediately used in the subsequent experiment.

### RNA-Seq on 4-Cell Embryos and Library Preparation

To identify global mRNA transcripts in 4-cell embryos, we prepared cDNA library with Single Cell Full Length mRNA-Amplification Kit (Vazyme, N712) following manufacturer’s instructions. In brief, embryos in M2 medium were washed in PBS and transferred to sample buffer for lysis. Then, reverse transcription of mRNA transcripts was conducted to obtain full length cDNA. Products of cDNA amplification were purified with Ampure XP beads (Beckman Coulter, A63881). Library preparation was performed with TruePrep™ DNA Library Prep Kit V2 for Illumina (Vazyme, TD503) following manufacturer’s instructions. After DNA quantification and qualification, all libraries were sequenced using NovaSeq, 6000 (Illumina, United States) by Beijing Novogene Bioinformatics Technology Co., Ltd., China.

### RNA-Seq Data Processing

After trimming, all RNA-seq reads were aligned to the mm10 genome using STAR v2.7.6a (Dobin et al., 2013; Bray et al., 2016). All unmapped reads, multi-mapped reads and PCR duplicates were removed. By using FeatureCounts (Liao et al., 2014), aligned reads were counted against gene model annotation (Gencode.vM27). Differentially gene expression analysis was performed with an R package DESeq2 v1.34.0 (Love et al., 2014) with negative binomial generalized linear models.

Then, we determined parent-of-origin of uniquely aligned reads with SNPsplit v0.5.0 (Krueger and Andrews, 2016), and then summarized all SNP-containing reads for each gene for allele-specific expression analyses. Likewise, differential gene expression analysis was computed with the DESeq2 R package v1.34.0. Only genes with RPKM > 1 in either group, *P*-value < 0.05, and FoldChange > 1.5 were considered as differentially expressed.

### Bioinformatic Analysis

We merged replicates of each group for downstream analysis, normalized read counts into RPKM by counting the numbers of reads in each 100-bp bin and then summed these counts within each 2-kb window for the entire genome using deeptools2 v3.4.3 (Ramírez et al., 2016). Genome-wide visualization of CUT&Tag and RNA-seq signals were conducted with the Integrative Genomics Viewer (IGV). Additionally, R packages ChIPseeker v1.28.3 (Yu et al., 2015) and clusterProfiler v4.0.5 (Yu et al., 2012) were used for annotation and Gene Ontology (GO) enrichment analysis, respectively.

## Results

### Cluster Characteristics of Integrated H3K4me3 and H3K27ac in CON Sperm

**FIGURE 1:**
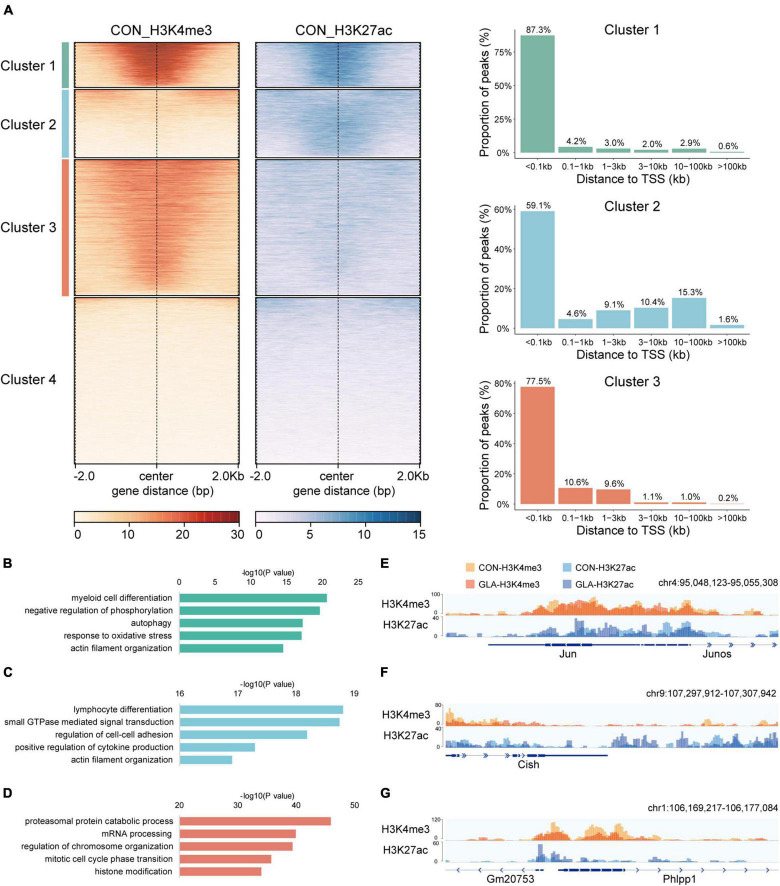
Cluster characteristics of integrated H3K4me3 and H3K27ac in CON sperm. **(A)** Heatmap (left) of k-means clustering of genome-wide H3K4me3 and H3K27ac signals ± 2.0 kb the center of H3K4me3 and H3K27ac regions in CON sperm, and bar graph (right) showing distribution of regions in Cluster 1–3 relative to the nearest transcription start site (TSS). For each cluster, genes were arranged in order of decreasing signals from top to bottom. **(B–D)** Selected significant pathways from Gene Ontology analysis on genes from Cluster 1 **(B)**, Cluster 2 **(C)** and Cluster 3 **(D)** in CON sperm. **(E–G)** Integrative Genomics Viewer tracks showing H3K4me3 and H3K27ac signals on *Jun* **(E)** from Cluster 1, *Cish* **(F)** from Cluster 2 and *Phlpp1* **(G)** from Cluster 3 in CON and GLA sperm.

At first, we conducted CUT&Tag on sperm of the CON group and integrated H3K4me3 and H3K27ac data to display their physiological patterns (Figure 1). We performed genome-wide k-means clustering of H3K4me3 and H3K27ac regions, and then divided them into 4 clusters. As shown in Figure 1A-left, Cluster 1 to Cluster 4 are characterized by both strong signals, weak H3K4me3 and strong H3K27ac, strong H3K4me3 and weak H3K27ac, and both weak signals, respectively. Then, we categorized peaks of the first three clusters by their distances to the nearest transcription start sites (TSS) (Figure 1A-right). In general, the majority of peaks were located less than 1 kb from TSS, while those with strong H3K27ac signals in Cluster 2 were also found over 10 kb from TSS in intergenic regions.

To enable functional interpretation of distinct patterns of H3K4me3 and H3K27ac, we annotated peaks in Cluster 1–3 to their nearest genes and identified biological pathways by GO enrichment analysis (Supplementary Table 1). Results showed that genes with overlapped H3K4me3 and H3K27ac signals were mainly enriched in pathways such as myeloid cell differentiation, negative regulation of phosphorylation and autophagy (Figure 1B). Those predominantly bearing abundant H3K27ac were enriched in pathways including GTPase signaling transduction and actin filament organization (Figure 1C). In addition, those with strong H3K4me3 signals in Cluster 3 were primarily enriched in mRNA and protein processing and chromosome organization (Figure 1D).

Next, we integrated H3K4me3 of CON and GLA sperm into one track, as well as H3K27ac of the two groups, and then zoomed in representative regions of these three clusters (Figures 1E–G). We found that overall GLA sperm had quite similar H3K4me3 and H3K27ac patterns with CON, while moderate intensity changes could still be observed in specific genomic loci such as decreased H3K4me3 in *Phlpp1* promoter of GLA sperm.

### Glufosinate-Ammonium-Induced H3K4me3 and H3K27ac Alterations in Mouse Sperm

**FIGURE 2:**
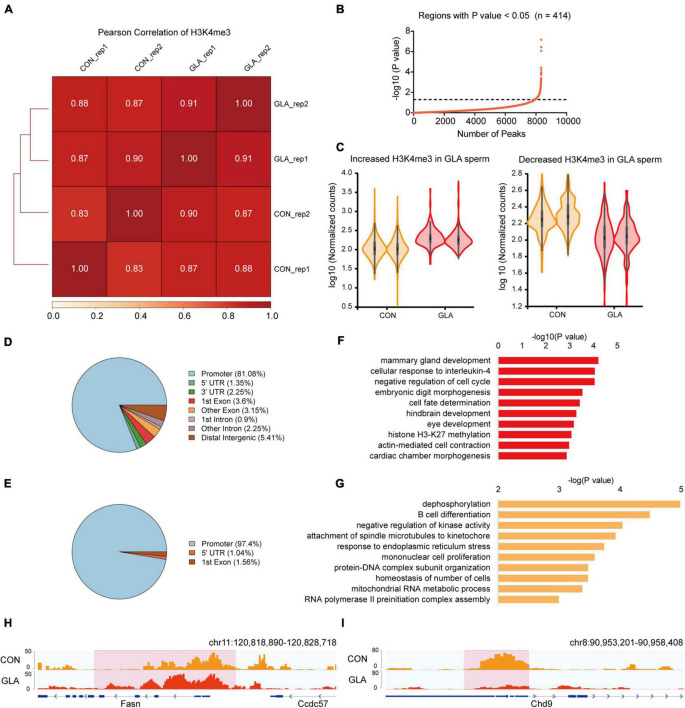
GLA-induced H3K4me3 alterations in mouse sperm. **(A)** Correlation matrix showing Pearson correlation coefficient of H3K4me3 between replicates of CON and GLA sperm. **(B)** Selection of regions with *P*-value < 0.05. The black dashed line corresponds to the boundary of *P*-value = 0.05. **(C)** Violin plots showing increased and decreased H3K4me3 in GLA sperm. Two replicates in each group are in the same color. **(D,E)** Pie chart showing the genomic distribution of regions with increased **(D)** and decreased **(E)** H3K4me3 signals in GLA sperm. **(F,G)** Selected significant pathways from Gene Ontology analysis on genes with increased **(F)** or decreased **(G)** H3K4me3 signals in GLA sperm. **(H)** Integrative Genomics Viewer tracks showing increased H3K4me3 on *Fasn* in GLA sperm. **(I)** Integrative Genomics Viewer tracks showing decreased H3K4me3 on *Chd9* in GLA sperm. Pink box indicates regions with differential H3K4me3 signals (*P*-value < 0.05).

To assess whether GLA exposure would alter global profiles of H3K4me3 and H3K27ac in mouse sperm, we further analyzed their sequencing data separately. As for H3K4me3, Pearson correlation analysis showed that the correlation coefficients of H3K4me3 between each two replicates of CON and GLA sperm were over 0.83 (Figure 2A). Then we compared H3K4me3 levels between two groups and found 414 significantly differential peaks with a *P*-value < 0.05 (Figures 2B,C and Supplementary Table 2). Increased H3K4me3 occupied regions were primarily distributed in promoters (81.08%) and distal intergenic regions (5.41%) (Figure 2D), while most of decreased H3K4me3 were also located in promoters (97.4%) (Figure 2E). According to results of GO enrichment analysis (Supplementary Table 2), genes like *Fasn* annotated by increased H3K4me3 were mainly enriched in biological pathways including cellular response to interleukin-4, hindbrain development and histone H3-K27 methylation (Figures 2F,H). For genes with decreased H3K4me3 levels, such as *Chd9*, were enriched in dephosphorylation, B cell differentiation, and RNA polymerase II preinitiation complex assembly related pathways (Figures 2G,I).

**FIGURE 3:**
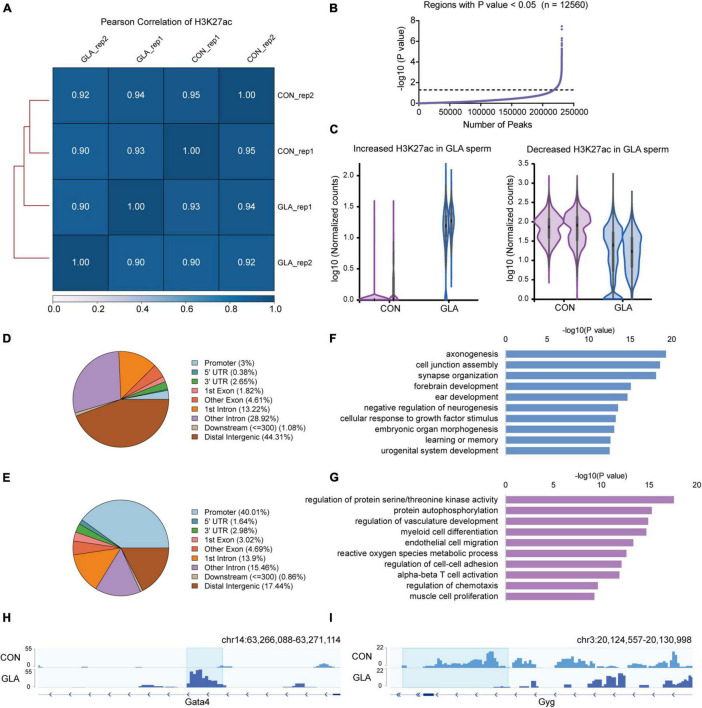
GLA-induced H3K27ac alterations in mouse sperm. **(A)** Correlation matrix showing Pearson correlation coefficient of H3K27ac between replicates of CON and GLA sperm. **(B)** Selection of regions with *P*-value < 0.05. The black dashed line corresponds to the boundary of *P*-value = 0.05. **(C)** Violin plots showing increased and decreased H3K27ac in GLA sperm. Two replicates in each group are in the same color. **(D,E)** Pie chart showing the genomic distribution of regions with increased **(D)** and decreased **(E)** H3K27ac signals in GLA sperm. **(F,G)** Selected significant pathways from Gene Ontology analysis on genes with increased **(F)** or decreased **(G)** H3K27ac signals in GLA sperm. **(H)** Integrative Genomics Viewer tracks showing increased H3K27ac on *Gata4* in GLA sperm. **(I)** Integrative Genomics Viewer tracks showing decreased H3K27ac on *Gyg* in GLA sperm. Blue box indicates regions with differential H3K27ac signals (*P*-value < 0.05).

Similarly, we did all these analyses on global H3K27ac level in sperm. All the Pearson correlation coefficients between each two replicates of CON and GLA sperm were over 0.90 (Figure 3A). In 12,560 differentially H3K27ac occupied regions (*P*-value < 0.05; Figure 3B and Supplementary Table 3), those with increased and decreased H3K27ac were analyzed separately (Figure 3C). Increased H3K27ac were mainly distributed in distal intergenic regions (44.31%) and introns (42.14%) (Figure 3D) while decreased H3K27ac were also primarily located in promoters (40.01%) and distal intergenic regions (17.44%) (Figure 3E). GO enrichment analysis (Supplementary Table 3) showed that gene with increased H3K27ac were mainly enriched in axonogenesis, synapse organization and learning or memory related pathways (Figure 3F), while those with decreased H3K27ac were enriched in pathways including protein autophosphorylation, myeloid cell differentiation, and regulation of chemotaxis (Figure 3G). IGV tracks showed representative deposition of increased H3K27ac in *Gata4* (*P*-value = 0.0038; Figure 3H) and decreased deposition in *Gyg* (*P*-value = 8.12 × 10^–7^; Figure 3I).

Taken together, GLA exposure alters H3K4me3 and H3K27ac profiles in mouse sperm, and their differentially occupied regions are distinct on genomic distribution and biological pathways.

### Differential Sperm mRNA Transcripts in Connection With H3K4me3 and H3K27ac Alterations in Glufosinate-Ammonium Sperm

**FIGURE 4:**
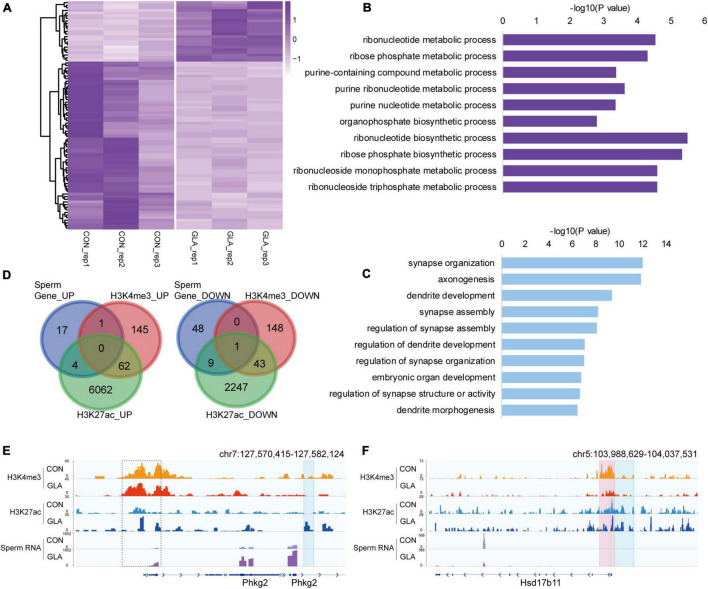
Differential sperm mRNA transcripts in connection with H3K4me3 and H3K27ac alterations in GLA sperm. **(A)** Heatmap of normalized mRNA transcripts in CON and GLA sperm (*n* = 10, data from Ma et al., 2021). **(B,C)** Selected significant pathways from Gene Ontology analysis on up-regulated **(B)** or down-regulated **(C)** genes in GLA sperm. **(D)** Venn diagram showing the number of regions with different mRNA transcripts levels in GLA sperm that overlap regions with altered H3K4me3 and H3K27ac levels. Left, up-regulated regions overlapping regions with increased H3K4me3 and H3K27ac. Right, down-regulated regions overlapping regions with decreased H3K4me3 and H3K27ac. **(E)** Integrative Genomics Viewer tracks showing up-regulated *Phkg2* integrating with H3K4me3 and H3K27ac signals in GLA sperm. **(F)** Integrative Genomics Viewer tracks showing down-regulated *Hsd17b11* integrating with H3K4me3 and H3K27ac signals in GLA sperm. Pink box indicates regions with differential H3K4me3 signals (*P*-value < 0.05). Blue box indicates regions with differential H3K27ac signals (*P*-value < 0.05). Dashed box indicates regions with slightly differential histone methylation signals.

To better comprehend the alterations happened in GLA sperm, we integrated and analyzed H3K4me3 and H3K27ac profiles with sperm RNA-seq data from our previous study (Ma et al., 2021). RNA-seq data showed that a majority of detectable mRNA transcripts were decreased in GLA sperm (Figure 4A). GO enrichment analysis indicated distinct biological pathways involving these differentially expressed genes, such as ribonucleotide biosynthetic and metabolic process in up-regulated pathways (Figure 4B) as well as synapse, axonogenesis and dendrite development in down-regulated pathways (Figure 4C). Given the positive correlation between these two active epigenetic marks and gene transcription, we hypothesized that GLA induced H3K4me3 and H3K27ac alterations would somewhat demonstrate those differentially expressed genes in sperm. Of the 22 up-regulated genes in sperm, only one overlapped with H3K4me3 increased regions, and 4 overlapped with H3K27ac increased regions, including *Phkg2* (Figure 4D-left). Then we zoomed our IGV plot of sperm H3K4me3, H3K27ac and mRNA tracks in the *Phkg2* locus, and found increased deposition of both H3K4me3 and H3K27ac (*P*-value = 0.0204) at its promoter, which was concordant with increased mRNA transcripts on the gene body (*P*-value = 6.97 × 10^–5^; Figure 4E). Similarly, of the 58 down-regulated genes, there were 10 overlapping with H3K27ac decreased regions, and none of them overlapped with H3K4me3 decreased regions alone (Figure 4D-right). Interestingly, there was one gene occupied by both decreased H3K4me3 and H3K27ac, namely *Hsd17b11*. We found markedly decreased H3K4me3 (*P*-value = 0.0034) and H3K27ac (*P*-value = 0.0428) were distributed at the promoter region of *Hsd17b11*, which might account for the significant decline of mRNA transcripts (*P*-value = 0.0006; Figure 4F). It suggested that differential sperm mRNA transcripts were linked to alterations of H3K4me3 and H3K27ac profiles in GLA sperm.

### Differential Gene Expression in 4-Cell Embryos in Connection With H3K4me3 and H3K27ac Alterations in Glufosinate-Ammonium Sperm

**FIGURE 5:**
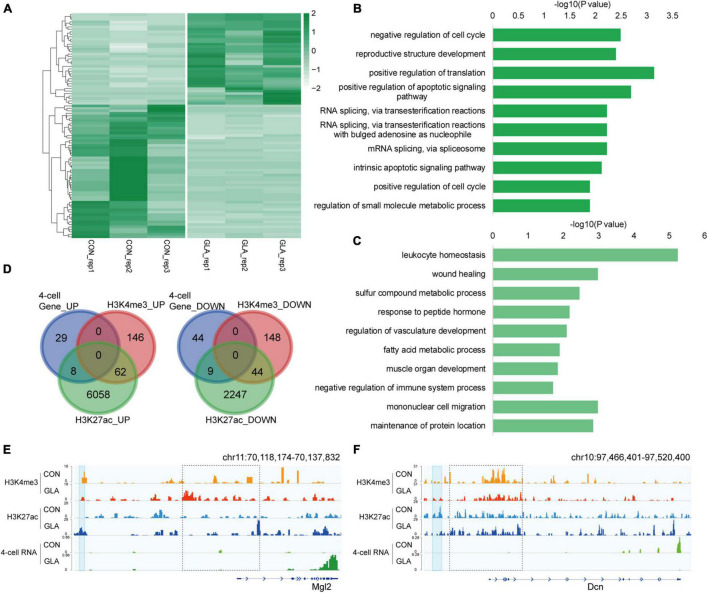
Differential gene expression in 4-cell embryos in connection with H3K4me3 and H3K27ac alterations in GLA sperm. **(A)** Heatmap of normalized gene expression levels in CON and GLA 4-cell embryos. **(B,C)** Selected significant pathways from Gene Ontology analysis on up-regulated **(B)** or down-regulated **(C)** genes in GLA 4-cell embryos. **(D)** Venn diagram showing the number of regions with different gene expression levels in GLA 4-cell embryos that overlap regions with altered H3K4me3 and H3K27ac levels in GLA sperm. Left, up-regulated regions overlapping regions with increased H3K4me3 and H3K27ac. Right, down-regulated regions overlapping regions with decreased H3K4me3 and H3K27ac. **(E)** Integrative Genomics Viewer tracks showing up-regulated *Mgl2* in 4-cell embryos integrating with H3K4me3 and H3K27ac signals in GLA sperm. **(F)** Integrative Genomics Viewer tracks showing down-regulated *Dcn* integrating with H3K4me3 and H3K27ac signals in GLA sperm. Blue box indicates regions with differential H3K27ac signals (*P*-value < 0.05). Dashed box indicates regions with slightly differential histone methylation signals.

Considering the contribution of paternal chromosome to the embryonic development, we hypothesized that GLA exposure induced sperm H3K4me3 and H3K27ac alterations might associate with aberrant gene expression in preimplantation embryos. Thus, we collected 4-cell embryos as a critical period after fertilization and performed RNA-seq to measure transcriptomic profiles in 4-cell embryos. It was revealed that 37 genes were significantly up-regulated and 53 genes were significantly down-regulated in GLA embryos (Figure 5A and Supplementary Table 4). GO analysis (Supplementary Table 4) showed that those up-regulated genes were mainly enriched in biological pathways including mRNA splicing and apoptotic signaling (Figure 5B), while down-regulated genes were predominantly enriched in leukocyte homeostasis and mononuclear cell migration related pathways (Figure 5C). Then we used Venn diagram to check whether GLA sensitive H3K4me3 and H3K27ac regions in sperm overlapped with those differentially expressed genes in 4-cell embryos. We found that of the 37 up-regulated genes, only 8 overlapped with increased sperm H3K27ac, including *Mgl2* (Figures 5D-left,E). Similarly, of the 53 down-regulated genes in 4-cell embryos, only 9 genes overlapped with decreased H3K27ac signals in sperm, such as *Dcn* (Figure 5D-right,F). The *Dcn* profile showed a significantly declined H3K27ac deposited at its putative enhancer (*P*-value = 0.0040), and H3K4me3 signals at the promoter were also reduced in sperm. Overall, these overlapping regions indicated that paternal GLA exposure caused differential gene expression in 4-cell embryos, which might be associated with H3K4me3 and H3K27ac alterations in GLA sperm.

### Differentially Parental-Origin Gene Expression in 4-Cell Embryos in Connection With H3K4me3 and H3K27ac Alterations in Glufosinate-Ammonium Sperm

**FIGURE 6:**
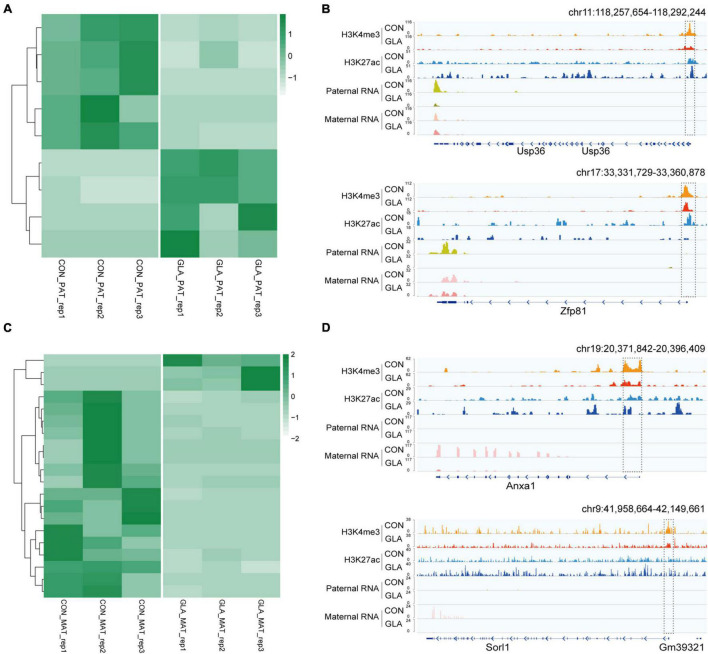
Differentially parental-origin gene expression in 4-cell embryos in connection with H3K4me3 and H3K27ac alterations in GLA sperm. **(A)** Heatmap of normalized mRNA transcripts of paternal alleles in CON and GLA 4-cell embryos. **(B)** Integrative Genomics Viewer tracks showing RNA transcripts of parental-specific alleles (*Usp36* and *Zfp81*) in 4-cell embryos integrating with H3K4me3 and H3K27ac signals in GLA sperm. **(C)** Heatmap of normalized mRNA transcripts of maternal alleles in CON and GLA 4-cell embryos. **(D)** Integrative Genomics Viewer tracks showing RNA transcripts of parental-specific alleles (*Anxa1* and *Sorl1*) integrating with H3K4me3 and H3K27ac signals in GLA sperm. Dashed box indicates regions with slightly differential histone methylation signals.

By identifying differential gene expression in 4-cell embryos induced by paternal GLA exposure, it aroused our interest on whether these differences occurred at the paternal or the maternal alleles. To figure out such the problem, we assigned RNA-seq reads to its parent-of-origin by examining their single nucleotide polymorphisms (SNPs) derived from C57BL/6J male mice or DBA/2 female mice. As shown in Figure 6A, there were 5 significantly down-regulated and 4 significantly up-regulated paternal-origin genes (*P*-value < 0.05; Supplementary Table 5). We integrated histone modifications in sperm with biparental gene expression in 4-cell embryos, and found genes like *Usp36* and *Zfp81* were significantly down-regulated on paternal allele (*Usp36*: *P*-value = 1.52 × 10^–5^; *Zfp81*: *P*-value = 1.89 × 10^–7^) and almost unchanged on maternal allele in GLA 4-cell embryos, which might be regulated by decreased H3K4me3 and H3K27ac at their promoters in sperm (Figure 6B). As for maternal-origin genes, only 3 of them were significantly up-regulated, and other 17 genes were significantly down-regulated (*P*-value < 0.05; Figure 6C and Supplementary Table 5). Accordingly, we zoomed in integrated tracks of these genes and found that both of the *Anxa1* and *Sorl1* kept paternally silenced but significantly down-regulated in maternal allele (*Anxa1*: *P*-value = 0.0001; *Sorl1*: *P*-value = 0.0003), which were concordant with decreased H3K4me3 and H3K27ac at their promoters in sperm (Figure 6D). Taken together, we found that differentially occupied H3K4me3 and H3K27ac in sperm induced by GLA exposure were linked to gene expression changes in both paternal and maternal alleles of 4-cell embryos.

## Discussion

As an emerging herbicide widespread in the environment, GLA and its metabolites have been detected in human biofluids such as serum (Aris and Leblanc, 2011) and urine (Bienvenu et al., 2021) samples, and it requires more evidence on its toxicity. In this study, we investigated genome-wide H3K4me3 and H3K27ac in sperm and transcriptome in preimplantation embryos to identify the potential hazard of GLA exposure, and integrated these sequencing data to find some clues on their association. Our study revealed that GLA exposure altered H3K4me3 and H3K27ac profiles in mouse sperm, which was linked to aberrant gene expression in preimplantation embryos.

Global profiles of sperm H3K4me3 and H3K27ac in CON mouse showed distinct distribution in genomic loci and biological pathways in each cluster. Sperm H3K4me3 is mainly distributed at developmental promoters (Hammoud et al., 2009), while H3K27ac is superior to identify super-enhancers (Hnisz et al., 2013) compared to other enhancer marks such as histone H3 lysine 4 monomethylation (H3K4me1), DNase hypersensitivity and p300. In this study, consistent with those in a physiological manner, differentially H3K4me3 and H3K27ac occupied regions in sperm induced by GLA exposure were mainly located at promoters and putative enhancers, respectively, which is concordant with their role as active promoter and enhancer marks (Calo and Wysocka, 2013). As for the number of differentially occupied regions, sperm H3K27ac seemed more sensitive than H3K4me3 to GLA exposure. According to GO analyses, both differentially occupied regions of sperm H3K4me3 and H3K27ac were enriched in biological pathways related to the immune system and nervous system, while those down-regulated genes in 4-cell embryos were also mainly enriched in immune-related pathways, indicating potential immune impairment in preimplantation embryos induced by paternal GLA exposure. Additionally, up-regulated genes in GLA sperm were primarily enriched in ribonucleotide biosynthetic and metabolic process. Given that glutamine is an indispensable substrate in ribonucleotide biosynthesis, we hypothesized that such pathway might be disturbed due to the structure similarity between glutamate and GLA.

Despite of some constantly changed genes in both sperm and embryos, most genes marked with active promoters in sperm are not expressed until the embryonic stage. For example, monovalent genes marked with H3K4me3 are mainly active in late spermatogenesis, while bivalent genes marked with H3K4me3 and H3K27me3 keep silence until embryonic development (Tomizawa et al., 2018). Though extensively inhibited, sperm retains the transcription complex composed of RNA polymerase II (RNAPII) and the mediator complex at about 60% promoters flanking nucleosomes deposited with H3K4me3 marks for immediate transcription after fertilization (Jung et al., 2019). Therefore, differentially occupied H3K4me3 and H3K27ac in sperm might be more related to gene expression changes in embryos than in sperm. At fertilization, protamine on paternal genome is immediately replaced by histones, and histone modifications are established followed by further reorganization in preimplantation embryos (Morgan et al., 2005). During that process, sperm H3K4me3 is erased in zygotes and then regained after zygotic genome activation (ZGA) in late 2-cell embryos in mouse (Zhang et al., 2016; Jung et al., 2019) and the mid-blastula transition (MBT) in zebrafish (Zhu et al., 2019). As reported by Lismer et al. (2020) altered H3K4me3 enrichment was inherited from sperm of the histone demethylase KDM1A transgenic mice to preimplantation embryos. In their subsequent study, diet-induced changes in sperm H3K4me3 were also transmitted into preimplantation embryos (Lismer et al., 2021). Additionally, a study comparing H3K4me3 profiles among sperm, preimplantation embryos and male gonad primordial germ cells showed that H3K4me3 marks within promoters of RNA splicing genes could escape two rounds of paternal reprogramming (Hao et al., 2021), which further proves the stability of H3K4me3 during the process of male reproduction and development.

When it comes to sperm H3K27ac, evidence becomes more limited. By immunostaining, the protein level of H3K27ac was similarly elevated both in sperm and embryos of zebrafish after paternal exposure to bisphenol A (BPA) (Lombó et al., 2019). Thus, it still needs further examination whether regions marked with H3K27ac in sperm could be inherited to embryos. Though we did not explore genome-wide profiles of H3K4me3 and H3K27ac in preimplantation embryos, transcriptome analysis indicated GLA altered embryonic gene expression. In addition, embryogenesis is a highly conserved process that involves cell fate decision. Many genes in early embryos have a dramatic impact on embryo development (Gardner and Lane, 2005; Leng et al., 2019) and offspring health (Shen et al., 2007; Zhao et al., 2013). Here we found that up-regulated genes in 4-cell embryos were mainly enriched in mRNA splicing related pathways, which reminded us the reprograming evasion of H3K4me3 marks mentioned above (Hao et al., 2021). By contrast, down-regulated genes were mostly enriched in immune related pathways. Therefore, such embryonic gene expression induced by paternal GLA exposure might threaten embryo development and offspring health, which requires further investigation.

Interestingly, we found differentially expressed genes in both paternal and maternal alleles of preimplantation embryos induced by paternal GLA exposure. Evidence reporting similar situation is quite limited even when it comes to lifestyle or other environmental stress, and its mechanism still remains unknown. Meanwhile, such differentially parental-origin gene expression caused non-canonical genomic imprinting in GLA embryos. For example, paternal GLA exposure newly generated a maternally expressed profile of *Zfp81* (Figure 6B), and made that of *Anxa1* disappeared (Figure 6D). Given to the crucial role of genomic imprinting in embryo and placenta development, fetus and neonate growth, as well as neurological behaviors and metabolism in adult offspring (Anckaert et al., 2013), differentially parental-origin gene expression in 4-cell embryos induced by GLA exposure might lead to unknown health risks in offspring developmental periods.

## Conclusion

By examining genome-wide profiles of sperm H3K4me3 and H3K27ac, together with embryonic gene expression, we concluded that GLA exposure altered sperm H3K4me3 and H3K27ac. Most of them were distributed at gene promoters and enhancers and enriched in the immune and nervous system related pathways. Additionally, GLA exposure disrupted biparentally immune-related gene expression in preimplantation embryos, which was associated with sperm H3K4me3 and H3K27ac profile changes. Though it still needs further investigation on whether these changes in sperm H3K4me3 and H3K27ac persist in preimplantation embryos, alterations in embryonic gene expression observed in our study sounded the alarm for potential adverse impact of paternal GLA exposure on embryo development and offspring health.

## Data Availability Statement

The datasets presented in this study can be found in online repositories. The names of the repository/repositories and accession number(s) can be found below: Genome Sequence Archive (Genomics, Proteomics, and Bioinformatics 2021); GSA: CRA005732.

## Ethics Statement

The animal study was reviewed and approved by the Institutional Animal Care and Use Committee (IACUC) of Nanjing Medical University (1811056–2).

## Author Contributions

XM: investigation, writing—original draft, reviewing, and editing. YF: software, formal analysis, and data curation. WX: investigation and validation. XD: investigation. WH: conceptualization, methodology, and supervision. YX: resources provision, funding acquisition, and supervision. All authors contributed to the article and approved the submitted version.

## Conflict of Interest

The authors declare that the research was conducted in the absence of any commercial or financial relationships that could be construed as a potential conflict of interest.

## Publisher’s Note

All claims expressed in this article are solely those of the authors and do not necessarily represent those of their affiliated organizations, or those of the publisher, the editors and the reviewers. Any product that may be evaluated in this article, or claim that may be made by its manufacturer, is not guaranteed or endorsed by the publisher.
